# A systematic review of controlled studies: do physicians increase survival with prehospital treatment?

**DOI:** 10.1186/1757-7241-17-12

**Published:** 2009-03-05

**Authors:** Morten T Bøtker, Skule A Bakke, Erika F Christensen

**Affiliations:** 1 Department of Anesthesiology and Intensive Care, Aarhus Hospital Nørrebrogade, University Hospital of Aarhus, Aarhus, Denmark

## Abstract

**Background:**

The scientific evidence of a beneficial effect of physicians in prehospital treatment is scarce. The objective of this systematic review of controlled studies was to examine whether physicians, as opposed to paramedical personnel, increase patient survival in prehospital treatment and if so, to identify the patient groups that gain benefit.

**Methods:**

A systematic review of studies published in the databases PubMed, EMBASE and Cochrane from January 1, 1990 to November 24, 2008. Controlled studies comparing patient survival with prehospital physician treatment vs. treatment by paramedical personnel in trauma patients or patients with any acute illness were included.

**Results:**

We identified 1.359 studies of which 26 met our inclusion criteria. In nine of 19 studies including between 25 and 14.702 trauma patients in the intervention group, physician treatment increased survival compared to paramedical treatment. In four of five studies including between nine and 85 patients with out of hospital cardiac arrest, physician treatment increased survival. Only two studies including 211 and 2.869 patients examined unselected, broader patient groups. Overall, they demonstrated no survival difference between physician and paramedical treatment but one found increased survival with physician treatment in subgroups of patients with acute myocardial infarction and respiratory diseases.

**Conclusion:**

Our systematic review revealed only few controlled studies of variable quality and strength examining survival with prehospital physician treatment. Increased survival with physician treatment was found in trauma and, based on more limited evidence, cardiac arrest. Indications of increased survival were found in respiratory diseases and acute myocardial infarction. Many conditions seen in the prehospital setting remain unexamined.

## Background

The scientific evidence for an effect of prehospital emergency medical services (EMS) on patient survival is limited and mainly based on case series and cohort studies [1,2]. As stated by Callaham [2]: "There is more solid scientific evidence about topics such as herbal medicine, acupuncture, hives, and constipation than there is about the entire practice of EMS."

Two previous reviews on the effect of advanced life support (ALS) vs. basic life support (BLS) found contradicting results on patient outcome [3,4]. A recent study found impaired survival with ALS compared to BLS in a subgroup of trauma patients with Glasgow Coma Score (GCS) < 9 [5]. However, these studies only included patients treated by paramedical personnel. Other studies have shown that physician treatment may increase survival and add life years for some groups of patients, especially patients with trauma, cardiac arrest and respiratory failure [6,7].

The closest any trial on the effect of prehospital treatment by physicians has come to a randomized, controlled design was published in 1987 [8]. The study examined blunt trauma patients receiving treatment by a paramedic/nurse or physician/nurse crew. Although dispatch of each crew was considered random, depending on rotation of calls or distance to scene, the design might carry inherent bias because differences in time to arrival to scene may influence the results. The study showed a decrease in mortality in the group treated by physicians. Since then, ethical considerations about informed consent have obstructed randomization – in Europe by laws and a directive [9], making controlled studies the highest possible level of evidence in this area.

The effect of physician based prehospital treatment using updated transport logistics has only been reviewed in combination with helicopter emergency medical services (HEMS) [10]. Therefore, the aim of this study was by means of a systematic review of controlled studies to examine whether physicians as opposed to other paramedical personnel increase patient survival in prehospital treatment and if so, to identify the patient groups that gain most benefit.

## Methods

Original, controlled studies comparing prehospital physician treatment with treatment by paramedical personnel were included. We required that treatment by physicians was an additional therapeutic intervention. We included studies with trauma-patients or patients with acutely developed known or unclear medical, surgical or psychiatric conditions or worsening of such. Studies using the outcome measures: survival, mortality or derivates were considered valid regardless of follow up time. Studies published in any language were included.

### Search strategy

Studies were identified by a search in the databases PubMed, EMBASE and Cochrane. The search strands are specified in additional file 1. In all databases, the search was limited to studies published from January 1, 1990 to November 24, 2008. In addition, we conducted a comprehensive search by the feature "related articles" in PubMed and through cross-references from already included original articles and from other reviews.

We systematically excluded studies not meeting the inclusion criteria in a hierarchical manner. First the title of a study, as it appeared from the search pages in the respective databases, was read and searched for the first of four predefined exclusion criteria: 1) Not prehospital treatment: studies that did not involve treatment of acutely ill patients out of hospital or any other medical institution, studies of organizational or safety-related issues in the prehospital setting and studies of inter hospital transfer of patients, 2) Not physicians: studies evaluating the effect of other health personnel, procedures or equipment, but not the effect of physicians, 3) Not controlled: all studies that did not explicitly compare a group of patients treated by physicians with a group of patients treated by paramedical personnel, including studies that compared physicians with paramedical personnel indirectly utilizing historical control studies and 4) Not survival: studies that did not use survival, mortality or derivates of these as outcome measures. If the study could not be excluded based on criteria 1, we searched for criteria 2 and so forth. If a study could not be excluded based on title, the abstract was searched. If exclusion could not be done based on the abstract, the entire article was searched. For each study we registered whether it was excluded by title, abstract or article and by criteria 1, 2, 3 or 4. Two reviewers (Bøtker MT and Bakke SA) conducted the searches in duplicate, and discrepancies regarding exclusion were solved by consensus with a third reviewer (Christensen EF). In cases of further doubt, whether a study was eligible for inclusion, an email was sent to the corresponding author of the article for clarification. The two reviewers independently extracted study details from the included articles. We predefined data to be extracted: study design, type and number of patients in each group, prehospital setting, raw survival data, adjustment and results. Discrepancies regarding data extraction were solved by consensus with the third reviewer.

### Study assessment

The studies were categorized according to study type: 1) controlled cohort studies, i.e. studies comparing outcome with different treatment over a period of time 2) system comparison studies, i.e. studies comparing outcome in two geographically separate areas and 3) before-and after studies, i.e. studies comparing outcome before and after a change in personnel. We ranked the studies in tables according to the number of patients in the intervention group: 1) less than 100 patients 2) 100–1000 patients and 3) more than 1000 patients. Afterwards we sorted them according to result: 1) studies demonstrating increased survival with physician treatment 2) studies not demonstrating a significant difference and 3) studies demonstrating decreased survival with physician treatment. A result was considered statistically significant if 95% confidence intervals were exceeded or p values were < 0.05.

For studies necessitating subgroup analysis, we attempted to assess, whether a subgroup analysis was intended a priori or whether it was a post hoc decision. Studies with subgroup analysis were initially categorized according to overall findings and ranked according to total number of patients. Afterwards, the results were categorized according to subgroup result and ranked according to the number of patients in the sub-intervention group. For some trauma studies using the TRISS methodology [11] based on Trauma Score [12] and Injury Severity Score (ISS) [13], interpretation was necessary to clarify whether the difference was significant or not. The Z statistic (± 1.96 required for significance) was utilized to describe the deviation in mortality (or survival) in a study group from the Major Trauma Outcome Study (MTOS) population [14] or the W statistic to describe how many more/less survivors than expected from the MTOS benchmark there were. In one study, CANALS – adjusting for variables like Revised Trauma Score, Injury Severity Score, age, sex, type of accident and type and number of treatment, was used [15].

## Results

### Literature search

Detailed results of the literature search are presented in Table 1. A total of 1.189 studies were identified searching PubMed. Of these, 128 were published in non-English languages. We identified 95 studies on EMBASE. Of these, ten were published in non-English languages. A total of 75 studies were identified searching Cochrane. Of these, three were published in non-English languages. From the 1.359 studies 1.318 were excluded according to the exclusion criteria defined above. From the remaining 41 studies an additional 18 studies were excluded due to database repetitions (n = 14) because physicians were involved in treatment of both intervention and control groups (n = 1) and because treatment by physicians could not be considered an additional intervention (n = 3). Using the PubMed feature "related articles" for the remaining 23 studies we identified an additional three studies [16-18] for inclusion. Cross-reference search prompted no additional studies for inclusion. Thus, a total of 26 studies were included [15-40]. One was published in German [26], the remaining in English. Due to significant influence for the interpretation of the result in the study by Nicholl et al. [32], a study by Younge et al., found by the original literature search, was included as a sub analysis [41]. None of the included studies were randomized.

**Table 1:** Results of the literature search

	**Number**	**Not prehospital treatment**	**Not physicians**	**Not controlled**	**Not survival**	**Included**
**Pubmed**	**1.189**	**507**	**473**	**161**	**17**	**31**
Title	936	489	365	75	7	0
Abstract	187	16	99	64	8	0
Article	66	2	9	22	2	31
						
**EMBASE**	**95**	**14**	**56**	**16**	**1**	**8**
Title	62	13	45	3	1	0
Abstract	21	1	9	11	0	0
Article	12	0	2	2	0	8
						
**Cochrane**	**75**	**23**	**50**	**0**	**0**	**2**
Title	66	23	43	0	0	0
Abstract	7	0	7	0	0	0
Article	2	0	0	0	0	2
						
**Total**	**1.359**	**544**	**579**	**177**	**18**	**41**

It is specified how many studies were excluded according to title, abstract and article. For each of these it is specified how many were excluded according to the predefined exclusion criteria.

Nineteen of the 26 studies only included trauma patients [15,17,21,22,24-37,40]. Five studies only evaluated patients with out of hospital cardiac arrest [16,18,23,38,39]. Two of the 26 studies included broader, unselected patient groups; all patients retrieved with helicopter [19] and all patients attended by an ambulance [20].

### Study assessment

An overview of the studies, presenting data as they were extracted from the articles, is presented in additional file 2. Of the 26 reviewed studies, 16 were categorized as "cohort studies" [15,17,21-26,29,31-34,37,39,40], five as "system comparison studies" [18,28,30,35,36] and five as "before and after studies" [16,19,20,27,38].

In five studies we used the result of a specified subgroup in our final assessment [18,20,24,29,40]. In only one of these studies it was specified that subgroup analysis was a priori intended [20]. The interpretation of two studies using TRISS needs further explanation. In the study by Nicholl et al [32], W statistical analysis showed more deaths than expected from the MTOS benchmark in both intervention and control group. Younge et al. pointed out, that the M statistic, describing match of injury severity between the study group and the MTOS population, was inappropriate due to a higher number of patients with high injury severity score in the study group [41]. Consequently, stratification according to probability of survival was required. Sub analysis using adjusted W (Ws) suggested a statistically significant 4.16 +/- 2.21 per 100 excess survivors in the intervention group. This result was included as a sub analysis of the study by Nicholl et al., and the result of the study was interpreted as an increase in survival with physician treatment. In the study by Schmidt et al [36], Z statistical analysis showed a significantly higher survival than expected in the intervention group and a trend towards higher survival in the control group compared with the historical MTOS control group. The two actual study groups were not compared directly. The result was interpreted as not significant.

### Survival

As presented in Table 2, of 26 included studies, 14 studies demonstrated a significantly higher survival in the intervention group than in the control group or in a relevant subgroup of these [15,16,20,23-25,31-33,35,37-40]. Nine studies did not show significant differences [17-19,21,22,27,28,34,36]. Three studies demonstrated a significantly lower survival in the intervention group than in the control group or any relevant subgroup of these [26,29,30].

**Table 2:** Results

Number of patients in intervention group	Physicians increase survival	Not significant	Physicians decrease survival
<100	Dickinson et al., 1997 (n = 9)	Suominen et al., 1998 (n = 49)*^2^	
	(Suominen et al., 1998 (n = 25))*^2^	Di Bartolomeo et al., 2005 (n = 56)	
	Soo et al., 1999 (n = 38 et 37)	Iirola et al., 2006 (n = 81)	
	Nardi et al., 1994 (n = 42)	Di Bartolomeo et al., 2001 (n = 92)	
	Garner et al., 1999 (n = 67)	(Mitchell et al., 1997 (n < 100)) *^1^	
	Sipria et al., 2000 (n = 70)		
	Frandsen et al., 1991 (n = 85)		
	Schwartz et al., 1990 (n = 93)		
100–1.000	(Frankema et al., 2004 (n = 103))*^3^	Frankema et al., 2004 (n = 107)*^3^	Graf et al., 1993 (n = 107)
	Osterwalder, 2003 (n = 196)	Hamman et al., 1991 (n = 145)	(Lee et al., 2003 (n = ?))*^6^
	Oppe et al., 2001 (n = 210)	Cameron et al., 2005 (n = 211)	Liberman et al., 2003 (n = 801)
	Mitchell et al., 1997 (n = 306)*^1^	Schmidt et al., 1992 (n = 221)	
	(Nicholl et al. 1995/Younge et al., 1997 (n = 337))*^4^	Lee et al., 2003 (n = 224)*^6^	
	(Christensen et al., 2003 (n = 177 et 388))*^5^	Ringburg et al., 2007 (n = 260)	
		Nicholl et al., 1995 (n = 337)*^4^	
>1.000	Roudsari et al., 2007 (n = 14.702)	Lechleutner et al., 1994 (n = 2.013)	
		Christensen et al., 2003 (n = 2.869)*^5^	

Studies are sorted according to overall result and ranked according to number of patients in the intervention group. Subgroup results are placed in parentheses and ranked according to number of patients in the sub-intervention group

*^1 ^Due to a significant difference in witnessed events and patients receiving bystander CPR, the authors made a sub analysis on patients with witnessed collapse, bystander CPR and presenting rhythm of VF/VT. In this group, only a trend towards increased survival was found – how many patients this group comprised was not given, but it was less than 100 in the intervention group.

*^2 ^Significantly higher survival in a group of patients with ISS from 25 to 49 – these comprised 51% (25/49) in the intervention group and 31% (22/72) in the control group

*^3 ^Significantly higher survival only in a subgroup of patients with blunt trauma – these comprised 82% (195/239) in the control group and 96% (103/107) in the intervention group

*^4 ^Not significant when calculated by Nicholl et al., later analysis using Ws by Younge et al. suggested higher survival.

*^5 ^Significant results only in subgroups of patients with AMI and respiratory diseases – these groups comprised 3% (177/5819) and 7% (388/5819) of the included patients

*^6 ^Significantly lower survival in a subgroup of patients not admitted to intensive care unit – this group comprised 50% of the included patients.

**Abbreviations: **See additional file 2

As presented in Table 3, of 19 studies in trauma patients, nine showed a significantly higher survival in the intervention group than in the control group or any relevant subgroup of these [15,24,25,31-33,35,37,40]. Of these, four studies included less than 100 patients in the intervention group or subgroup where significant differences were detected [25,31,37,40], four included between 100 and 1.000 [15,24,32,33] and one study included more than 1.000 patients [35]. Additional data on this study was extracted from another article by the same first author [42]. The study included 14.702 patients in the intervention group and showed a lower mortality (OR = 0.7 (CI 0.54 – 0.91)) among severely injured patients with ISS > 15 in countries with prehospital ALS by physicians than in countries with ALS by paramedics or technicians. For patients with more severe injuries, (ISS > 25) this finding was even more pronounced; OR = 0.57 (CI 0.39 – 0.73). Seven of the studies in trauma patients did not show significant differences [17,21,22,27,28,34,36]. Of these, three studies included less than 100 patients in the intervention group or subgroup demonstrating significant differences [21,22,27], three included between 100 and 1.000 [17,34,36] and one included more than 1.000 [28]. Three studies demonstrated a significantly lower survival in the intervention group than in the control group [26,29,30]. All of these studies included between 100 and 1.000 patients in the intervention group or subgroup demonstrating significant differences.

**Table 3:** Results in trauma patients

Number of patients in intervention group	Physicians increase survival	Not significant	Physicians decrease survival
<100	*(Suominen et al., 1998 (n = 25))*	Di Bartolomeo et al., 2005 (n = 56)	
	Nardi et al., 1994 (n = 42)	Iirola et al., 2006 (n = 81)	
	Garner et al., 1999 (n = 67)	Di Bartolomeo et al., 2001 (n = 92)	
	Schwartz et al., 1990 (n = 93)		
100–1.000	*(Frankema et al., 2004 (n = 103))*	Hamman et al., 1991 (n = 145)	Graf et al., 1993 (n = 107)
	Osterwalder, 2003 (n = 196)	Schmidt et al., 1992 (n = 221)	*(Lee et al., 2003 (n = ?))*
	Oppe et al., 2001 (n = 210)	Ringburg et al., 2007 (n = 260)	Liberman et al., 2003 (n = 801)
	*(Nichool et al./Younge et al., 1997)*		
>1.000	Roudsari et al., 2007 (n = 14.702)	Lechleutner et al., 1994 (n = 2.013)	

For studies with subgroup analysis only subgroup results are displayed

As presented in Table 4, four of five studies in patients with out of hospital cardiac arrest revealed a significantly higher survival in the intervention group than in the control group [16,23,38,39] and one did not show significant differences [18]. All studies included less than 100 patients in the intervention group or subgroup demonstrating significant differences.

**Table 4:** Results in patients with out of hospital cardiac arrest

Number of patients in intervention group	Physicians increase survival	Not significant	Physicians decrease survival
<100	Dickinson et al., 1997 (n = 9)	*(Mitchell et al., 1997 (n < 100))*	
	Soo et al., 1999 (n = 38 et 37)		
	Sipria et al., 2000 (n = 70)		
	Frandsen et al., 1991 (n = 85)		
100–1.000			
>1.000			

For studies with subgroup analysis only subgroup results are displayed

The study by Christenszen et al. of unselected patients attended by an ambulance included 2.869 patients in the intervention group and showed no difference in overall survival. However, a significantly higher long-term survival for a subgroup of patients with acute myocardial infarction (AMI) (n = 177) and a significantly higher short-term survival for a subgroup of patients with respiratory diseases (n = 388) were demonstrated [20]. The study by Cameron et al., of unselected patients retrieved by helicopter, did not show significant differences. In this study, 211 patients were included in the intervention group [19].

## Discussion

Few studies met our inclusion criteria. In the two groups most studied – trauma patients and patients with out of hospital cardiac arrest – an improved survival with physician treatment was found. Indications that survival might be increased for other patient groups were found, but an increase in survival could not be demonstrated for broader, unselected patient groups attended by EMS.

We found no randomized controlled studies. Because this was suspected, we had chosen to include controlled studies a priori. This was done in order to pursue the best available evidence, in line with the intention of evidence based medicine [43]. However, we only found 26 controlled studies for inclusion and many of the studies included very few patients. The studies were inhomogeneous and as a consequence we did not perform a true meta-analysis.

The risk of publication bias is a well-recognized limitation of systematic reviews [44]. We sought to minimize this by including studies in other languages than English in order to avoid bias introduced by the tendency to publish very unique results in an English journal and otherwise in a journal of native language. The large number of studies not fulfilling our inclusion criteria demonstrates the degree of difficulty in constructing a concise search in this area. This is mainly caused by a huge variability in the terms used for physicians, emergency services and transport platforms in the prehospital setting. It increases the risk that includable studies were missed by the original search. In addition, there was a considerable difference between the number of studies located in PubMed, Cochrane and EMBASE. This is probably caused by our rigorous search method that was initially intended for PubMed. We intended to counteract these limitations by an initial systematic search in all three databases and subsequently by systematically using the "related articles" function in the PubMed database and searching cross-references in articles from already included studies.

One of the confounding factors that may influence the results was transportation, i.e. physicians were transported by helicopter, the other crew by ground ambulance [15,21,22,24,26,27,31-34,37,40]. However, the confounding influence may only be minor because the studies demonstrated an increase as well as a decrease and no change in survival suggesting absence of any systematic bias. The observation is in accordance with the fact that the effect on survival with helicopter transport has not yet been clarified [7,45]. Lossius et al [6] estimated that the major part of life years gained could be explained by treatment and only ten percent by fast helicopter transport. Consequently, we made no attempt to adjust for transportation logistics. Another potential confounder is the merging of comparisons between not only differences between prehospital physician vs. non-physician treatment but also between other differences in system logistics [18,28,30,35,36] and comparison of survival before and after a larger reorganization [38].

In 14 studies, an increased survival with physician treatment was demonstrated. One of these studies on trauma patients was large-scaled and well designed system comparison study [35]. In particular three other studies were appropriately designed [20,25,31]. One of these was a system comparison study [25], another included few patients in the intervention group [31], reducing the strength of this study, but one was large-scaled and found an increased survival in a priori defined subgroups [20]. In three studies finding increased survival with physician treatment, the strength was limited by a low number of patients in the intervention group [23,39,40] and two studies had obvious confounding factors because only physicians carried defibrillators [16] and because the addition of physician treatment was part of other organizational improvements [38]. Three studies revealed a reduced survival with physician treatment. In one of these, a higher ISS and a lower GCS in the intervention group than in the control group remained unadjusted for [26]. In another, the patients were divided into subgroups depending on admittance to ICU or not and it was not specified why this division was chosen [29]. Hence, the result should be interpreted with caution. One of the studies finding a decrease in survival with physician treatment was well-designed [30] and possible explanations for the impaired survival with ALS found in some studies are prolonged on-scene time in patients requiring in-hospital definitive treatment (e.g. patients with penetrating trauma) and suboptimal endotracheal intubation [4]. Nine studies did not demonstrate any significant difference in survival. In particular three studies were well designed, but all included a limited number of patients in the intervention group or relevant subgroup [18,21,22] increasing susceptibility to type 2 errors. One of these examined patients in cardiac arrest after blunt trauma [22]. These patients have a very serious prognosis and an expected survival of nearly zero. It should be noted that survivors (n = 2) were only found in the group treated by physicians. In one small study, exclusion of patients dying in the ambulance induced a risk of selection bias [27]. In one study survival rates were very high in both intervention and control group (97.2% and 97.5%) reflecting that for patients with an a priori low risk of dying, physicians do not increase survival [19].

Thus, the quality and strength of the evidence supporting an increase in survival with physician treatment was variable and could be influenced by publication bias. However, many of the studies not finding a significant difference in survival were susceptible to type 2 errors and the evidence supporting a decrease in survival was very sparse and mostly of questionable quality. Hence, the results are encouraging – in particular for the most studied group – trauma patients. Since trauma patients and patients with out of hospital cardiac arrest were the only specified groups studied, many conditions seen in the prehospital setting remain unexamined. Future research should address this aspect.

It was beyond the scope of our review to consider that the only two studies analyzing admission rates, demonstrated that physicians were able to complete treatment of more patients on site and thus avoid unnecessary hospital admission [19,20]. Avoided admissions may be of importance not only for the individual patient, but also have potential socioeconomic implications. This is an obvious topic for future research.

## Conclusion

A systematic review revealed only few controlled studies examining survival with prehospital physician treatment. The quality and strength of the included studies was variable and many conditions remain unexamined. Increased survival with physician treatment was found in the groups most extensively studied – trauma patients and, based on a more limited evidence, patients with cardiac arrest. Indications of increased survival were found in respiratory diseases and acute myocardial infarction.

## Competing interests

The authors declare that they have no competing interests.

## Authors' contributions

MTB conceived and designed the study, carried out one duplicate of the search and study details extraction, and drafted the manuscript. SAB carried out the other duplicate of the search and study details extraction, and helped drafting the manuscript. EFC participated in conceiving the study, participated in its design and revised the manuscript. All authors have read and agreed to the content.

## Supplementary Material

Additional file 1 **Appendix 1. **Search strands Click here for file

Additional file 2 **Appendix 2**. Study overview Click here for file
