# Supplementary material for: A systematic review of controlled studies: do physicians increase survival with prehospital treatment?
Source: Scand J Trauma Resusc Emerg Med. 2009 Mar 5;17:12. doi: 10.1186/1757-7241-17-12 (PMC2657098; doi:10.1186/1757-7241-17-12)
Supplement: Additional file 1 — Appendix 1. Search strands [file 1757-7241-17-12-S1.doc]

# Appendix 1 – Search strands

In PubMed and Cochrane, the following search strand was used: (anesthetist OR anesthesiologist OR physician OR doctor OR case OR advanced life support OR helicopter) AND (paramedic OR nurse OR emergency medical technician OR control OR basic life support OR ambulance) AND (prehospital OR pre-hospital OR helicopter OR out-of-hospital OR emergency medical services[MESH]) and (outcome assessment OR survival OR mortality). In PubMed, the limit “humans” was used. In Cochrane, the search strand was used searching “Title, Abstract or Keywords”.

In EMBASE, the following search strand was used: (anesthetist OR anesthesiologist OR physician OR doctor OR case OR advanced life support OR helicopter) AND (paramedic OR nurse OR emergency medical technician OR control OR basic life support OR ambulance) AND (prehospital OR pre-hospital OR helicopter OR out-of-hospital OR emergency medical services) AND (outcome assessment OR survival OR mortality). In EMBASE, the limit “humans” was used.
