# Supplementary material for: A systematic review of controlled studies: do physicians increase survival with prehospital treatment?
Source: Scand J Trauma Resusc Emerg Med. 2009 Mar 5;17:12. doi: 10.1186/1757-7241-17-12 (PMC2657098; doi:10.1186/1757-7241-17-12)
Supplement: Additional file 2 — Appendix 2. Study overview [file 1757-7241-17-12-S2.doc]

**Appendix 2.** Study overview – studies divided by study type and listed by publication year

| Study | Patients | Setting | Survival | Adjusted | Results |
| --- | --- | --- | --- | --- | --- |
| **Controlled cohort studies** | | | | | |
| Ringburg AN et al., 2007  [34]  The Netherlands | Trauma  Intervention: 260  Control: 1.197 | Intervention: attended by physician/EMT staffed HEMS  Control: attended by nurse/paramedic staffed EMS ambulance | 1 month  Intervention: 75.8 %  Control: 95.5% | Yes | Intervention vs. control. Mortality OR = 1.0 (CI 0.8-1.3) |
| Di Bartolomeo et al., 2005  [22]  Italy | Blunt trauma, found in cardiac arrest  Intervention: 56  Control: 73 | Intervention: attended by physician staffed daytime helicopter  Control: attended by nurse staffed ambulance | To hospital discharge  Intervention: 3.5%  Control: 0.0% | No | Difference NS (CI -0.008 - 0.078) |
| Frankema et al., 2004  [24]  The Netherlands | Trauma  Intervention: 107  Control: 239 | Intervention: attended by physician staffed helicopter  Control: attended by paramedic staffed ambulance | 2 years  Intervention: 65.4%  Control: 75.7% | Yes | Intervention vs. control. Survival OR = 2.2 (0.92 - 5.9)  SG: Blunt trauma, OR = 2.8 (CI 1.07 - 7.52) |
| Osterwalder JJ, 2003  [33]  Switzerland | Blunt trauma  Intervention: 196  Control: 71 | Intervention: attended by physician staffed helicopter/ambulance  Control: attended by EMT/paramedic/nurse staffed ambulance | 30 days  Intervention: 88.8%  Control: 85.9% | Yes | Control vs. intervention. Mortality OR = 37 (CI 2 - 749) |
| Lee A et al., 2003  [29]  Australia | Blunt trauma  Intervention: 224  Control 1: 1.167  Control 2: 497 | Patients were divided into those admitted to the ICU and those not (no-ICU)  Intervention: attended by physician staffed ambulance  Control 1: attended by paramedic staffed ambulance  Control 2: attended by EMT staffed ambulance  Patients (n = 96) transported by non-EMS were left out in this review. | To hospital discharge  Intervention: 80.4 %  Control 1: 75.2 %  Control 2: 87.7 % | Yes | Intervention vs. control 2, No-ICU. Mortality OR = 4.27 ( CI 1.46 - 12.45)  Intervention vs. control 2, ICU. Mortality OR = 0.63 (CI 0.28 - 1.39) |
| Di Bartolomeo et al., 2001  [21]  Italy | Severe head injury  Intervention: 92  Control: 92 | Intervention: attended by physician staffed daytime helicopter  Control: attended by nurse staffed ambulance | To hospital discharge  Intervention: 69.6 %  Control: 76.1 % | No | Intervention vs control. Mortality OR = 1.39 (CI 0.72 – 2.67) |
| Oppe S et al., 2001  [15]  The Netherlands | Trauma  Intervention: 210  Control: 307 | Intervention: attended by physician staffed helicopter  Control: attended by EMT staffed ambulance | 15 months  Intervention: 72.4%  Control: 75.9% | Yes – CANALS as described in [15] | Lives saved by intervention = minimum 11 – 17 (p < 0.05) |
| Soo LH et al., 1999  [39]  UK | Out of hospital cardiac arrest  Intervention 1: 70  Intervention 2: 56  Control: 520 | Comparison between many different personnel. Of 2094 patients, many control groups were left out. EMT staffing was used as index.  Intervention 1: attended by paramedic/physician staffed ambulance  Intervention 2: attended by EMT/physician staffed ambulance  Control: attended by EMT staffed ambulance | To hospital discharge  Intervention 1: 15.7 %  Intervention 2: 8.9 %  Control: 4.4 % | Yes | Intervention 1 vs. control. Survival OR = 20.88 (CI 6.72 - 64.94)  Intervention 2 vs control. Survival OR = 3.24 (CI 1.03 – 10.20) |
| Garner A et al., 1999  [25]  Australia | Blunt trauma.  Intervention: 67  Control: 140 | Intervention: attended by physician staffed helicopter  Control: attended by paramedic staffed helicopter | To hospital discharge  Intervention: 85.1 %  Control: 80.7 % | TRISS as described in [11] | Ws = 13.33 (CI 7.80 - 19.08) in favor of intervention group |
| Suominen P et al.,  1998  [40]  Finland | Pediatric patients with blunt trauma  Intervention: 49  Control: 72 | Intervention: attended by physician staffed helicopter/ambulance  Control: attended by EMT staffed ambulance | Not specified  Intervention: 77.6 %  Control: 68.1 % | No | Difference NS  SG: Patients with ISS 25-49: higher survival in intervention group (p < 0.05) |
| Dickinson ET et al., 1997  [23]  USA | Out of hospital cardiac arrest  Intervention: 9  Control: 40 | Intervention: attended by physician staffed ambulance  Control: attended by paramedic staffed ambulance | To hospital discharge  Intervention: 44 %  Control: 5 % | No | Difference significant (p < 0.009) |
| Nicholl JP et al., 1995  [32]  UK | Trauma  Intervention: 337  Control: 466 | Intervention: attended by physician staffed helicopter  Control: attended by paramedic staffed ambulance | 6 months  Intervention: 72.7 %  Control: 83.5 % | TRISS | W statistic showed 16% more deaths than predicted in intervention group vs. 2% more deaths than predicted in control group |
| Nardi G et al., 1994  [31]  Italy | Trauma  Intervention: 42  Control 1: 82  Control 2: 98 | Intervention: attended by physician staffed helicopter and directly transported to trauma center  Control 1: attended by nurse staffed ambulance, stabilized at nearest hospital and transferred to trauma center  Control 2: attended by nurse staffed ambulance and directly transported to trauma center | To ICU discharge  Intervention: 88.1 %  Control 1: 62.2 %  Control 2: 68.4 % | No | Difference between intervention and control 1 (p < 0.005) and between control 2 (p < 0.05) significant. |
| Graf M et al., 1993  [26]  Switzerland (In German) | Trauma Intervention: 107 Control: 131 | Intervention: attended by physician staffed helicopter  Control: attended by paramedic staffed ambulance | To hospital discharge Intervention: 79 % Control: 90% | No | The mortality was significantly higher in the intervention group (p = ?) |
| Hamman Bl et al., 1991  [17]  USA | Trauma  Intervention: 145  Control: 114 | Intervention: attended by physician staffed helicopter  Control: attended by nurse and/or paramedic staffed helicopter | Not specified  Intervention: 91.7 %  Control 93.0% | TRISS with mortality based Z statistic | Z, intervention = -2.03.  Z, control = -3.11.  Difference not significant |
| Schwartz RJ et al., 1990  [37]  USA | Blunt trauma  Intervention: 93  Control: 33 | Intervention: attended by physician staffed helicopter  Control: attended by paramedic staffed ambulance | Not specified  Intervention: 72.0 %  Control: 72.7 % | TRISS with survivor based Z statistic | Z, intervention = 2.23 Z, control = -2.69  Significantly higher survival in intervention group |
| **System comparison studies** | | | | | |
| Roudsari SB et al., 2007  [35]  Multiple countries | Trauma  Additional data extracted from [42] Intervention: 14.702  Control: 15.060 | Comparison between seven countries  Intervention: attended in one of four countries with physician operated emergency medical systems  Control: attended in one of five countries without physicians, but with technician operated ALS | No crude data | Yes | Intervention vs. control. Mortality OR = 0.7 (CI 0.54 – 0.91) |
| Liberman M et al., 2003  [30]  Canada | Trauma  Intervention: 801  Control 1: 1.000 Control 2: 7.604 | Comparison between two cities in the same country  Intervention: attended by physician in first city  Control 1: attended by paramedic in second city  Control 2: attended by EMT in any of the cities | To hospital discharge  Intervention: 65 %  Control 1: 76 %  Control 2: 82 % | Yes | Intervention vs. control 1. Mortality OR = 1.20 (CI 0.89 - 1.63)  Intervention vs. control 2. Mortality OR = 1.36 (CI 1.14 – 1.65) |
| Mitchell RG et al., 1997  [18]  UK/USA | Out of hospital cardiac arrest  Intervention: 306  Control: 723 | Comparison between two cities  Intervention: attended in system with physicians  Control: attended in system with paramedics | To hospital discharge  Intervention: 12.4 %  Control: 7.2 % | Yes | Difference significant (p < 0.01).  SG: witnessed collapse, bystander CPR and presenting rhythm VF/VT Intervention: 32.8 % vs. control: 15.6 % (NS) |
| Lechleutner A et al., 1994  [28]  Germany/USA | Trauma  Intervention: 2.013  Control: 1.153 | Comparison between two cities in two different countries  Intervention: attended in system with physicians  Control: attended in system with paramedics | No crude survival data | Yes | No difference (p = ?) |
| Schmidt U et al., 1992  [36]  Germany/USA | Trauma  Intervention: 221  Control: 186 | Comparison between two cities in two different countries  Intervention: Attended by physician staffed helicopter in first city  Control: Attended by nurse/paramedic staffed helicopter in second city | Not specified  Intervention: 90.5 %  Control: 88.7 % | TRISS with survivor based Z statistic | Z statistic, intervention group = 2.459 (significant), control group = 1.049 (NS) |
| **Before and after studies** | | | | | |
| Iirola et al., 2006  [27]  Finland | Blunt trauma  Intervention: 81  Control: 77 | Intervention: attended by physician staffed helicopter/ambulance after addition of this  Control: attended by EMT staffed ambulance before the addition | 3 years  Intervention: 69.1 %  Control: 81.8 % | No | Difference NS (p=0.065) |
| Cameron et al., 2005  [19]  Australia | All retrieved patients  Intervention: 211  Control: 163 | Intervention: attended by physician staffed helicopter after addition of this  Control: attended by paramedic staffed helicopter before the addition | 30 days  Intervention: 97.2 %  Control: 97.5 % | Yes | Difference NS (p = 0.817) |
| Christenszen EF et al., 2003  [20]  Denmark | All ambulance users  Intervention: 2.869  Control: 2.950 | Intervention: attended by EMT (72.3 %) or physician (27.7 %) staffed ambulance after addition of physicians on separate ambulances.  Control: attended by EMT staffed ambulance before the addition | 180 days  Intervention: 89.5%  Control: 90% | Yes | Intervention vs control. Mortality OR = 1.06 (NS) SG: AMI, OR = 0.2 (p < 0.001) |
| Sipria A et al., 2000  [38]  Estonia | Out of hospital cardiac arrest  Intervention: 70  Control 1: 40  Control 2: 61 | Intervention: attended after addition of physicians on separate ambulances, ACLS training of EMTs and resuscitation standards  Control 1: attended by EMT staffed ambulance with low-grade training and equipment before reorganization  Control 2: attended during reorganization | To hospital discharge  Intervention: 25.7 %  Control 1: 7.5 %  Control 2: 11.5 % | No | Difference between intervention and control 1 significant (p < 0.05) |
| Frandsen F et al., 1991  [16]  Denmark | Out of hospital cardiac arrest  Intervention: 85  Control 1: 160  Control 2: 148 | Intervention: attended in 2nd period by physician staffed ambulance  Control 1: attended in 1st period by EMT staffed ambulance  Control 2: attended in 3rd period by EMT capable of defibrillation staffed ambulances | To Hospital discharge  Intervention: 13 %  Control 1: 5 %  Control 2: 1 % | No | Difference between intervention and both control 1 and 2 significant (p < 0.001) |

Study: first author, publication year and country in which the study was conducted

Patients: type of patients and number of patients in intervention and control group

Setting: prehospital setting and unit/staff attending intervention and control group

Survival: follow up time and raw survival data

Adjusted: adjusted for patient characteristics such as presenting blood pressure, pulse, injury severity etc.

Results: results – in case the result was not statistically significant, significant results of a relevant subgroup was extracted

**Abbreviations:**

EMT: Emergency Medical Technician

HEMS: Helicopter Emergency Medical Service

NS: Not Significant

SG: Subgroup analysis

ICU: Intensive Care Unit

CANALS: An adjustment tool, adjusting for variables like Revised Trauma Score, Injury Severity Score, age, sex, type of accident and type and number of treatment(s)

TRISS: A method for evaluating trauma care, accounting for Trauma Score and Injury Severity Score

Ws: Stratified W statistic, i.e. how many more/less survivors than expected from the Major Trauma Outcome Study benchmark there was, stratified according to probability of survival

ISS: Injury Severity Score

Z: Statistic to describe the deviation in mortality (or survival) in a study group from the Major Trauma Outcome Study population. A Z value of more than ±1,96 is required for significance

CPR: Cardiopulmonary resuscitation

VF/VT: Ventricular Fibrillation / Ventricular Tachycardia

AMI: Acute Myocardial Infarction
